# Antibacterial properties of bimetallic nanopattern induced by excimer laser on PTFE nanotextile

**DOI:** 10.1016/j.heliyon.2025.e42775

**Published:** 2025-02-18

**Authors:** Petr Slepička, Tereza Vašinová, Bára Frýdlová, Anna Kutová, Šárka Havlíčková, Václav Švorčík, Nikola Slepičková Kasálková

**Affiliations:** Department of Solid State Engineering, The University of Chemistry and Technology Prague, 166 28, Prague, Czech Republic

**Keywords:** Bimetallic nanopattern, Noble metal, Polymer, PTFE, Nanotextile, Antibacterial properties, Replication, Nanostructure, Laser exposure

## Abstract

The construction of functional micro- or nanostructured surfaces is extensively studied since they are able to provide multifunctional properties and for large variety of potential applications in fields such as tissue engineering, wearable electronics or microfluidics. The micro- or nanosized surfaces can be easily prepared by various lithography techniques, also additional modifications (laser exposure, metal deposition and further processing) and which can induce new applicable properties on the basis of synergic effect by combining aforementioned approaches. In this work we have focused on the polytetrafluoroethylene (PTFE) nanotextile with specific bimetallic nanostructures. Our primary target was to find optimal surface modification of silver/gold coated surface, which would induce strong antibacterial response to both gram-positive and/or gram-negative bacteria. We have used plasma-modified polytetrafluoroethylene nanotextile as a substrate, onto which silver and gold nanolayers were deposited by sputtering. The foils were further subjected to "single-shot” exposure to an excimer KrF laser and some samples were also thermally stressed before exposure. Such surfaces were further examined in terms of surface morphology and chemical composition. The surface was investigated for antibacterial properties. Their antimicrobial activity was examined *in vitro* against the bacteria *Escherichia coli* and *Staphylococcus epidermidis* strains. The surface of the prepared materials was replicated into a lactic acid polymer and the properties were again investigated in terms of surface morphology and surface chemistry. The results demonstrated construction of antibacterial surfaces with excellent resistance to bacteria *E. coli* for bimetallic structures on PTFE. Excimer laser induced bimetallic pattern exhibited also significant antibacterial properties for *S. epidermidis*. Replication of bimetallic pattern was also demonstrated.

## Highlights

• Heat treatment and excimer laser was used for PTFE nanotextile enhancement.
•Bimetallic globular pattern (AuAg) was prepared on PTFE nanotextile.
•Proposed surface treatment led to antibacterial enhancement.
•Possibility of large scale manufacturing of treated substrates was suggested.

## Graphical abstract

**Figure:**
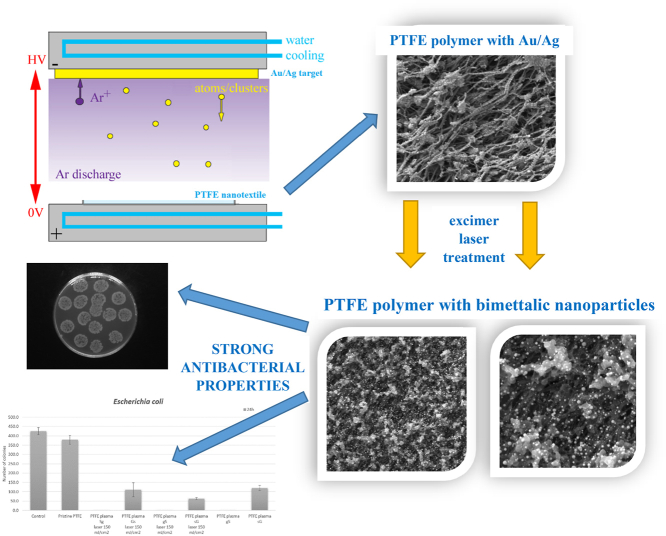

## 1 Introduction

Since their discovery, the use and development of fluoropolymers continues to grow, PTFE, polyvinylidene fluoride (PVDF) [1,2], fluorinated rubber materials (FKM) and fluorinated ethylene propylene (FEP) [3] are the most applicable materials. Fluorinated polymers are used in the construction industry (coatings resistant to UV radiation and pollution), petrochemical and automotive industries [4] or aerospace due to their resistance to extreme temperatures [5]. They are often applied as protective coatings of various types of materials (metals, wood, leather, textiles), and are also used as materials suitable for the creation of photoresistors, conductive polymers, biomaterials or in microlithography [6,7]. The most interesting and most used property of PVDF is its piezoelectricity, it is also pyroelectric and also ferroelectric substance [5], which is also highly stable against thermal stress and UV radiation [8]. It is used in robotics, various devices for medicine or acoustics, as well as in "energy harvesting” devices [9].

The only bonds found in the polymer are the bonds between carbons C-C and between carbon and fluorine C-F, which are covalent in nature and in both cases they are very strong bonds. Fluorine atoms are significantly larger than the original hydrogen atoms, thus ensuring not only chemical stability and steric protection, but also other properties such as non-stickiness, low surface energy and low friction coefficient [10]. The PTFE molecule is relatively simple and symmetrical and has a good ability to organize into crystalline structures, between which there are amorphous regions. The ability to crystallize is related to another property of the polymer: the melting point. Higher crystallinity (92–98 %) increases its value, and PTFE therefore mostly melts at 320–342 °C. During melting, it reaches very high densities, which makes common processes such as injection molding very difficult and much more complex and expensive process techniques (e.g. sintering) must be used [11]. From a biological point of view, it is also very stable, resisting the body's environment as well as enzymatic or microbiological processes in the body [12]. It is considered physiologically inert and non-toxic up to temperatures of 260 °C. At higher temperatures, it begins to release vapours into its surroundings, and at temperatures higher than 350 °C it can cause poisoning to exposed persons. Pyrolysis and decomposition back into monomeric units occurs at temperatures of 400 °C [13].

PTFE as a biomaterial was first used to manufacture an artificial heart valve. As one of the few synthetic materials, it can be stable in the human environment (up to 6.5 years) [14]. It is used in the form of a textile or its "expanded” version (ePTFE). Both molds contain pores, the size of which can be regulated during production. Porosity can be an advantage: it causes tissue ingrowth into the implant and also the formation of an endothelial layer. On the other hand, however, other unwanted substances can also easily adhere, which can cause clinical problems [15]. The advantage of the expanded form of the polymer is also its non-thrombogenic surface [16]. It is beneficial in implants for contact with blood, oxygenating devices or filters, where there is a risk of adsorption of blood proteins and clinical complications [17]. Other products are metal stents coated with ePTFE designed to prevent the growth of tumors and benign formations. Coated catheters are used to drain urine after excretory tract operations, and there are also microporous PTFE balloons that deliver drugs to targeted tissues in the body [18]. In orthopedic surgery, we encounter this material during surgical replacement of ligaments in joints, it is also used for a relatively short time in soft tissue replacements in plastic and reconstructive surgery [19]. In tissue engineering, nanofibrous materials made from biocompatible polymers can be used as substitutes for skin, bones, heart, nerve and other tissues. The high porosity resembles the structure of the extracellular matrix, which promotes cell adhesion, proliferation and differentiation. Nanofibrous membranes have been shown to be beneficial as wound dressings. In addition, due to the large surface area, keratinocytes are able to migrate along the structure, which subsequently accelerates healing. Membranes can also be used for local dosing of therapeutics such as antibiotics or growth factors [20]. As already mentioned, PTFE is also produced in the form of a textile or a porous membrane. Fluoropolymers such as PTFE or FEP have been also used in tissue engineering as an effective substrates for preparation of honeycomb structures from secondary polymers by improved phase separation technique [[21], [22], [23], [24], [25], [26], [27]].

Bimetallic nanoparticles have been frequently researched materials in recent years, as they have unique optical, electrical, magnetic and also catalytic properties. The advantage is the possibility to use the synergistic properties of both types of metals and to achieve specific desired properties by optimizing the composition, shape and type of the resulting nanoparticles. There are several types of bimetallic nanoparticles according to the arrangement of metal atoms [28]. Similar to nanoparticles composed of one type of metal, bimetallic nanoparticles can be prepared by three basic processes: chemically, physically, and biologically. Physical methods can include, for example, laser ablation, grinding or electrochemical processes, chemical productions include various types of reduction, precipitation or thermal decomposition, many biological methods can be used, for example microbial synthesis [29]. Typically, nanoparticles are prepared by pulsed laser deposition, where a colloidal solution of one type of material is first created by ablation from the target. Subsequently, the target is replaced and, by ablation, atoms of the second element of the metal are introduced into the already formed solution. The properties of the resulting particles can be influenced by the wavelength of the laser used, the type of stabilization medium and also the type of ablated metal [30]. The application possibilities of bimetallic nanostructures extend to several industries, such as automotive, aviation, energy, nuclear or medical [31]. In medicine, they can be used for diagnostics, cancer therapy, or for the production of systems for the targeted application of drugs. E.g. Au-Fe or Ni-Co nanostructures can be used as a contrast medium for CT and MRI analysis. Bimetallic nanoparticles are also advantageous materials for the production of nanosensors, nanochips and nanosemiconductors. The possibility of use in the chemical industry as reaction catalysts is also very important [29]. A common example is the combination of two noble metals: silver and gold. This type of nanostructures shows interesting antimicrobial applications. Silver nanoparticles are highly toxic to bacteria due to the release of Ag + ions into the environment. This gave them the prerequisite for *in vivo* use in the medical field. However, toxicity to certain types of mammalian cells has also been found, due to which promising *in vivo* applications have been rejected. However, if the silver nanoparticles were replaced by Au/Ag bimetallic structures, the release of Ag^+^ ions would be mitigated and biocompatibility increased [30]. Lin et al. [32] tested different ratios of Ag and Au in bimetallic nanoparticles and demonstrated that the composition Au40Ag60 reduces the cytotoxic effect while maintaining the antimicrobial properties.

Especially the development of antimicrobial surfaces has risen to the forefront of interest, as a possible solution for ongoing antibiotic-resistance crisis. Many recent studies were focused on development of alternative approach to prevent bacterial attachment by creating micro and nano-textured surface structures, inspired by naturally occurring antibacterial and antibiofouling surfaces, as a promising way of passive bacterial infection control in biomedical field. Combination of nanostructured surfaces and gold/silver is promising surface alteration approach resulting in synergic action and enhanced antibacterial performance. Previously we have applied the excimer laser treatment for coloration of aromatic polymers, e.g. the gold nanoparticles we fabricated on the polyethersulphone substrate [33]. The construction of silver nanocluster array was realized by the combination of heat treatment and the plasma pre-treatment of PTFE nanotextile [34]. Similar procedures was proposed earlier by Schmidl et al. [35] for construction of bi-metallic Pd/Au nanoparticles, the same groups prepared also porous nanostructures by laser [36]. The novelty of the presented study consists of development of simple fabrication method of nanostructured PTFE surfaces with bimetallic pattern. Specific approach of combination of high energy excimer laser and heat pre-treatment led to formation of bimetallic nanocluster array with strong antibacterial properties.

## 2 Experimental

### 2.1 Materials

#### 2.1.1 PTFE nanofibrous textile

Polytetrafluoroethylene polymer film was purchased from GoodFellow Ltd. (UK). This film is 0.045 mm thick with a pore size of 2 μm. The film has a matte white colour and is made of thin fibers, which determines its porous nature. According to the manufacturer, PTFE foil has good resistance to all types of solvents (acids, bases, alcohols, aromatic compounds, halogens, ketones, oils). Young's modulus was determined to be 0.3–0.8 GPa and tensile strength 10–40 MPa. The density is 2.2 g/cm^3^. It has good resistance to UV radiation, on the contrary, poor resistance to radiation. The working temperature is given by an interval from −260 °C to temperatures of 180–260 °C.

#### 2.1.2 PLLA film

Poly-L-lactic acid film was purchased from GoodFellow Ltd. (UK). This film is 0.05 mm thick and has a transparent colour. The density of the material is 1.25 g/cm^3^. The glass transition of PLLA occurs in the temperature range of 55–65 °C. The melting point is in the range of 145–160 °C. The material is classified by the manufacturer as a biodegradable biopolymer.

#### 2.1.3 Metal targets

Au and Ag targets for sputtering were purchased from Safina a.s. (Czech Republic). The company claims a purity of at least 99.99 %.

### 2.2 Plasma exposure

Before sputtering the metal particles, the surface of the PTFE foils was modified by the plasma modification method. The BAL-TEC Sputter Coater SDC 050 device was chosen for etching with argon plasma, which was set to the "etching” program. The set power was 8 W and etching was carried out for 240 s at a pressure of 10 Pa.

### 2.3 Sputtering

The surface treatment was followed by the deposition of films using the sputtering technique. The Quorum Q300T ES machine in the "timed sputter” program was chosen. Sputtering took place in a vacuum at a pressure of 1 Pa. The aim of this step was to prepare silver and gold layers of different thicknesses in variable orders. With reference to the article [37], a constant current of 60 mA was chosen and the variable variable was the sputtering time, for which two intervals were chosen: 100 s and 300 s. All possible combinations were prepared according to the following table Table 1.

**Table 1:** Variations of prepared samples with different combinations of gold and silver layers.

	Sputtering time [s]	
	**S (*silver*)**	**G (*gold*)**
**sg**	**100**	**100**
**gs**	**100**	**100**
**Sg**	**300**	**100**
**gS**	**300**	**100**
**sG**	**100**	**300**
**Gs**	**100**	**300**
**SG**	**300**	**300**
**GS**	**300**	**300**

For further description, symbols will always be used: "s" for silver and "g" for gold. A small letter (s/g) symbolizes a shorter sputtering time (always 100 s), a capital letter (S/G) then a longer duration (always 300 s). A parameter characterizing the process, i.e. sputtering time, not thickness, is used to differentiate the layers. Silver and gold particles have different ability to nucleate on the substrate, and at the same time, growth is conditioned by the properties of the substrate, and thus also by the thickness or continuity of any primary metal layer.

The samples were heated for 1 h at 100 °C. A Binder ED56 dryer was used.

### 2.4 Excimer laser exposure

PTFE foils with metal combinations of metal layers were exposed to a "single shot” pulse of a KrF excimer laser that induces radiation with a wavelength of 248 nm (Coherent Leap 100). The radiation energy used was 150 mJ/cm^2^ or 200 mJ/cm^2^ and was measured with a FieldMaxII-TOP device. The angle of the incident beam was 0°. The exposure was carried out in an atmosphere at room temperature.

### 2.5 Analytical methods

#### 2.5.1 Atomic force microscopy

Surface morphology and roughness of the pristine and treated films were examined by atomic force microscopy (AFM) technique using Dimension ICON (Bruker Corp., Billerica, MA, USA). The samples were analyzed in Scan-Assyst® mode using nitride lever SCANASYST-AIR with Si tip (spring constant of 0.4 N m^−1^). NanoScope Analysis software was applied for data processing. Surface roughness (*R*_*a*_) represents the arithmetic mean of the absolute values of the height deviations measured from the central plane.

#### 2.5.2 Scanning electron microscopy

The morphology of the samples surfaces was also characterized by complementary technique using the scanning electron microscope FIB-SEM LYRA3 GMU (Tescan. Brno, Czech Republic). The acceleration voltage was set to 10 kV. To ensure the conductivity of the samples, their metallization was performed using sputtering technique (Quorum Q300T) by deposition of Pt layer (thickness of 20 nm, Pt target, purity of 99.9995 %).

The elemental composition was measured by energy-dispersive X-ray spectroscopy (EDS, analyzer X-ManN, 20 mm^2^ SDD detector, Oxford Instruments, United Kingdom), while the accelerating voltage for SEM-EDS analysis was set to 10 kV.

#### 2.5.3 Wettability

Wettability of the studied samples was determined by measurement of contact angles (CA, θ) on goniometer Advex Instruments (Brno, Czech Republic) connected to the SEE System 7.1 program. Analysis of CA was performed at room temperature with 8 μL drops of distilled water (dyed with methyl violet) using a Transferpette® automatic pipette (Brand, Wertheim, Germany) at 6 different positions of 3 samples in parallel and perpendicular direction. Subsequently, the drops were photographed and evaluated by 3 marked points.

#### 2.5.4 Infrared spectroscopy

Absorption spectra were measured using PerkinElmer instrument, Lambda 850+ spectrometer type. The spectrometer has wavelength range of 190–1100 nm and is therefore suitable for both solutions and solid samples.

#### 2.5.5 Laser confocal microscopy

The surface morphology of the sample was also monitored by laser confocal microscopy on an Olympus LEXT OLS 5100 confocal scanning microscope. A GaN laser diode with a wavelength of 405 nm was used. Among the advantages of confocal microscopy is the possibility to place samples directly on the microscope stage - no vacuum is needed.

### 2.6 Antibacterial study

The antibacterial activity of the samples was determined using a drop test using gram-negative bacteria *Escherichia coli* and gram-positive bacteria *Staphylococcus aureus*. From agar plates of *E. coli* bacterial strains, one colony was transferred to 20 ml of liquid Luria-Bertani (LB) medium, from agar plates of *S. aureus* bacterial strains, one colony was transferred to 5 ml of LB medium. The inocula thus prepared were subsequently cultured overnight at 37 °C in an orbital shaker. The following day, the bacteria were diluted in sterile PBS to a concentration of approximately 1.6 × 10^3^ bacteria per 1 ml for *E. coli* and to a concentration of 8 × 10^4^ bacteria per 1 ml for *S. aureus*. The tested samples were placed in triplet on 2 ml of bacterial suspension with the modified side and were statically incubated at laboratory temperature. After 2 (respectively 5) and 24 h, the bacterial suspension was mixed and five drops of each with a volume of 25 μl were pipetted onto LB agar culture dishes. These plates were cultured overnight at 28 °C for *E. coli* and at 37 °C for *S. aureus*. Subsequently, the number of colony forming units (colony forming unit (CFU)) was determined, which was compared with the number of CFU in the control (bacteria incubated only in phosphate buffer under the same conditions). The experiment was performed under sterile conditions.

## 3 Results and discussion

PTFE porous foils were plasma modified at a power of 8 W for 240 s. The modification leads to a change in the chemical composition, fluorines from the surface are replaced by oxygen atoms and the O/C ratio increases and F/C decreases. This fact leads to a decrease in the contact angle and a decrease in the hydrophobic character of the material [38]. The treatment serves to ensure better adhesion of metal particles to the substrate [39]. Combinations of silver and gold layers on the textile were deposited according to Table 1. The selected samples were thermally stressed at 100 °C for 1 h. Subsequently, the materials were exposed to the KrF excimer laser at energy of 150 and 200 mJ/cm^2^. The surface of selected sample types was replicated into PLLA foils using the "hot-embossing” method.

### 3.1 Wettability

**Fig. 1:**
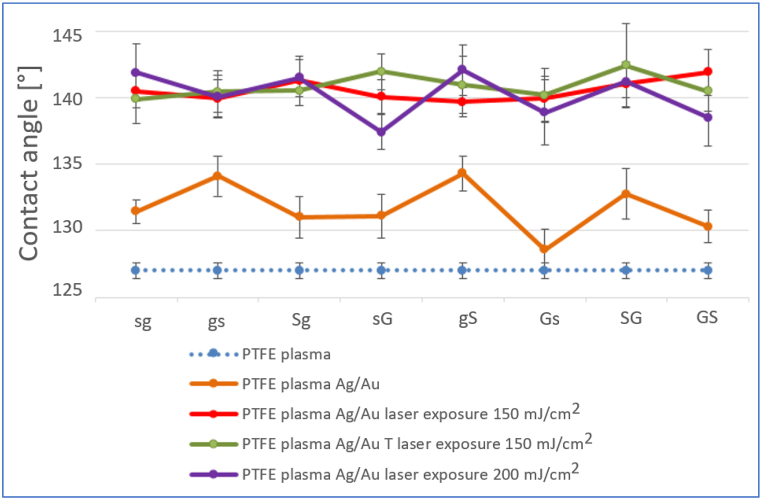
Comparison of the results of goniometric measurement for individual types of samples PTFE plasma with Ag/Au layers, PTFE plasma with Ag/Au layers after exposure to the laser (150 and 200 mJ/cm^2^) and PTFE plasma with Ag/Au layers and a combination of thermal stress techniques (1 h, 100 °C) and laser exposure (150 mJ/cm^2^).

Differences can be found between the wettability of different types of PTFE [40], e.g. the contact angle of the PTFE foil was determined to be 108° in the work [41]. The value of the contact angle of the pristine PTFE nanotextile film determined by me was 131.1 ± 0.8°. In the case of hydrophobic materials, the value of the contact angle also increases with increasing roughness [42]. There is a case where the value of the contact angle of a porous PTFE foil membrane was measured up to 141.9° [43]. The contact angle of the PTFE foil after plasma modification was also measured. The determined value was 127.0 ± 0.6°. The plasma modification resulted in a slight reduction in the contact angle, and thus a reduction in the hydrophobic character of the film. Sputtering of metal layers did not significantly change the wettability of the material (see Fig. 1). After the metal layer is deposited, the two effects are likely to be combined - a slight increase in material roughness and at the same time a change in surface chemistry. The expected value of the contact angles of distilled water on the surfaces of noble metals is around 60° [44], and metal layers should therefore reduce the value of the contact angle. The thickness of the layers and the order of the deposited metals do not have a significant effect on the wettability, since a continuous layer formation probably does not occur during sputtering and the hydrophobic nature of the substrate still prevails (Fig. 1).

For all samples exposed to the laser, the wettability decreased, in most cases the value even exceeded 140°. The change can be caused by the clustering of the metal film into nanoparticles, or the formation of PTFE-metal composite structures, at the same time there is a change in the surface morphology (see the following chapter) of the exposed PTFE substrate and thus an increase in the hydrophobic character. The same effect was also observed in article [37].

### 3.2 Surface morphology

The effect of the thickness and order of the metal layers on the surface morphology and the effect caused by exposure to KrF laser at different energy values (150 mJ/cm^2^ and 200 mJ/cm^2^) or the effect of a combination of thermal stress and laser exposure techniques were investigated.

**Fig. 2:**
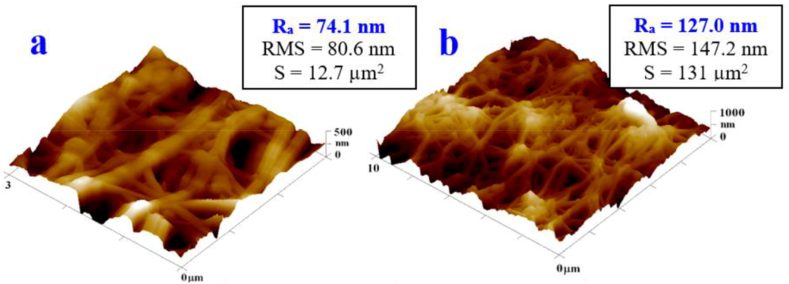
AFM images of unmodified PTFE foil 3 × 3 μm^2^ (left) and 10 × 10 μm^2^ (right) [37].

Fig. 2a and b shows AFM images of a pristine PTFE nanofibrous foil. The images were taken from the article [37], where the strong inertness of PTFE itself against KrF laser exposure was also demonstrated.

**Fig. 3:**
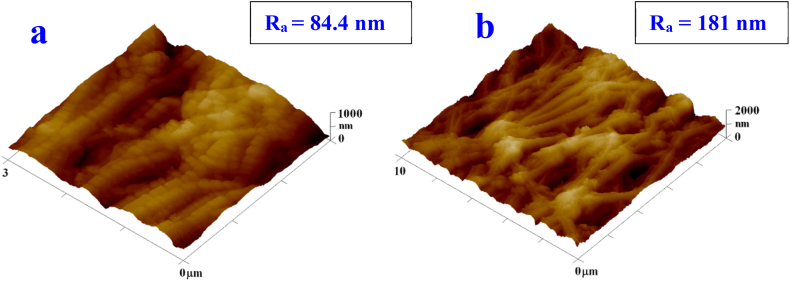
AFM images of plasma-modified PTFE foil (8 W, 240 s) 3 × 3 μm^2^ (a) and 10 × 10 μm^2^ (b).

In Fig. 3 we can see AFM images of plasma-modified PTFE. An increase in surface roughness and a change in surface morphology can be observed by exposure to Ar plasma. The first types of samples examined were foils with combinations of silver and gold layers (specifically sG, i.e. primary silver sputtered for 100 s and secondary gold for 300 s and gS, i.e. gold deposited for 100 s and then silver for 300 s). Compared to pristine PTFE, there was a change in the morphology of the fibers, which affected the wettability results, especially in combination with the influence of the deposited layers of noble metals. The structure of the foil in the form of nano/microfibers is preserved.

**Fig. 4:**
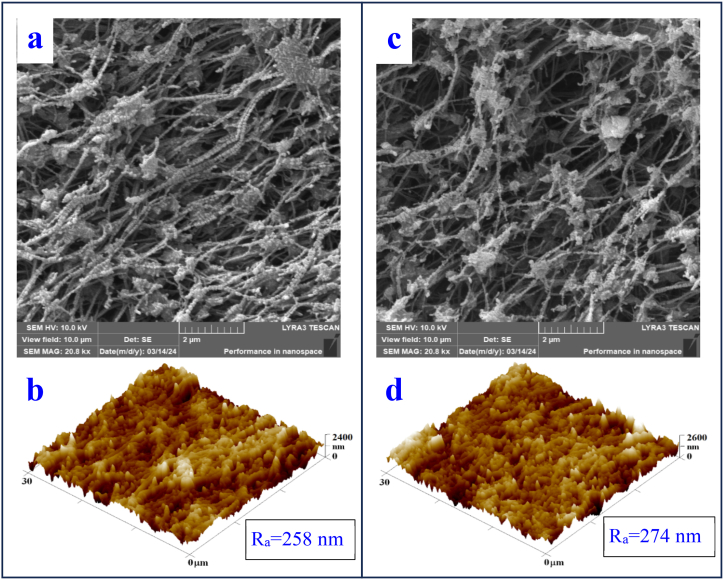
AFM (b, d) and SEM (a, c) images of plasma-modified PTFE foils covered with metal layers sG (a and b) and gS (c and d), (AFM area 30 × 30 μm^2^, SEM area 10 × 10 μm^2^). R_a_ represents the surface roughness value.

We also focused on samples with metal layers after laser treatment at an energy of 150 mJ/cm^2^. The images show the same combinations of sG and gS noble metal layers. After exposure to the laser, the fibrillar character of the substrate is preserved, a significant change in morphology occurs. Spherical bimetallic nanostructures and microstructures were formed and PTFE expanded to form a PTFE-noble metal composite (Fig. 4), while excimer laser exposure caused an significant change in surface roughness. No dramatic change in morphology has been seen between the different combination (sG and gS) of metal layers. The same conclusion was evaluated for the whole set of 8 samples (combination of Au and Ag – layer position and deposition time 100 s and 300 s) and for this reason further comparison of the morphology change within this set of samples will not be included.

**Fig. 5:**
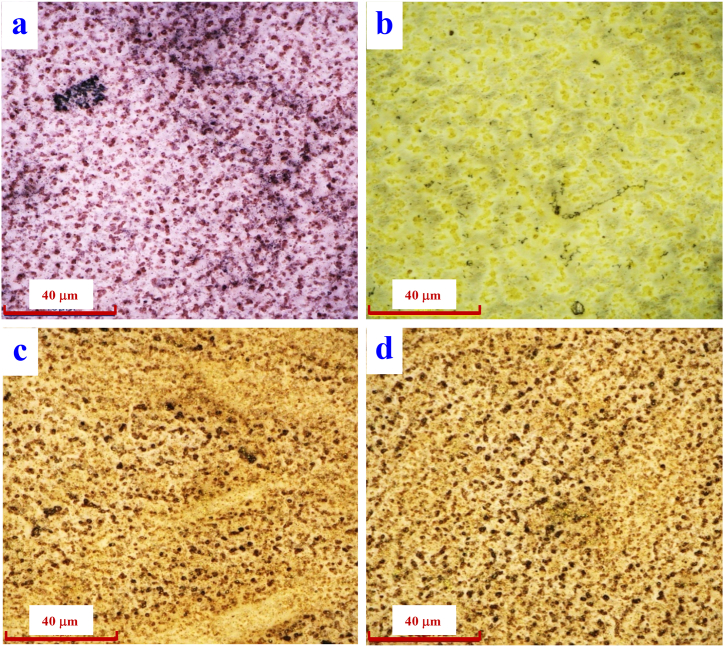
LCM microscopy image: PTFE plasma (a), gold layer after laser exposure (150 mJ/cm^2^), (b), silver layer after laser exposure (150 mJ/cm^2^), bottom combination of sg (c) and gs (d) metals after exposure laser (150 mJ/cm^2^), (displayed area 340 × 256 μm^2^).

According to Ref. [45], spherical silver nanoparticles with a small size distribution have a yellow colour. In turn, gold nanoparticles were investigated by Pahm et al. [46] who demonstrated the purple colour of gold nanoparticles in a polymer substrate. Based on the above knowledge, it is possible to assume the creation of an array of nanoparticles after laser exposure of layers of noble metals on PTFE. Therefore, the same phenomenon can be assumed for their combination, i.e. that the system of two layers of Au and Ag after exposure with an excimer laser will create a system of nanoparticles and metal-PTFE composite particles. Based on the colour of the structure created after laser exposure of the combination of these metals (Fig. 5), it can be said that these are not layers of individual substances on top of each other, but rather a composite of both elements. The same phenomenon was also observed in article [47] and was explained by the change of surface plasmon resonance during the formation of bimetallic structures.

**Fig. 6:**
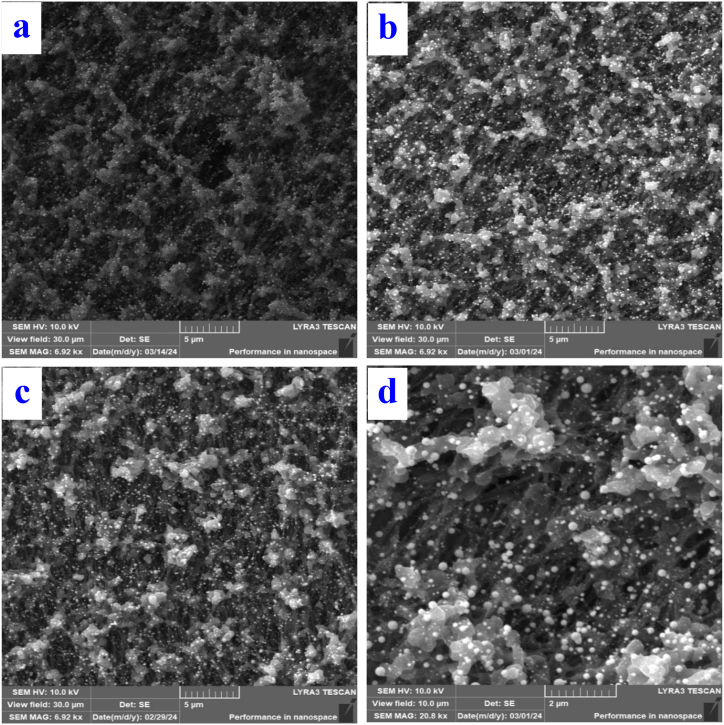
SEM images of PTFE plasma samples with sG metal layers (A) exposed to a laser with an energy of 150 mJ/cm^2^ (30 × 30 μm^2^), (B) after heating for 1 h at 100 °C and subsequent exposure to a laser with an energy of 150 mJ/cm^2^ (30 × 30 μm^2^), (C) exposed to a laser with an energy of 200 mJ/cm^2^ (30 × 30 μm^2^) and (D) exposed to a laser with an energy of 200 mJ/cm^2^ (10 × 10 μm^2^).

Furthermore, the effect of laser fluence and the combination of heating and laser exposure on the resulting structures on a PTFE plasma substrate covered with a sG layer was compared, see Fig. 6a–d. The samples did not show a significant morphological change, in all cases globular nanoparticles were formed on the surface of the PTFE fabric.

### 3.3 Surface chemistry - EDS

The chemical composition was studied using the EDS analysis. Plasma-modified PTFE samples covered with metal films and subsequently laser-exposed were investigated.

**Fig. 7:**
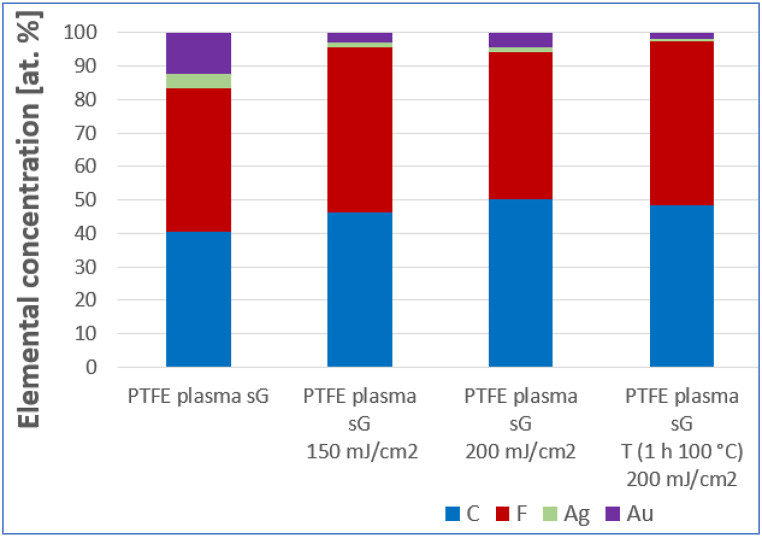
EDS analysis results: atomic concentration of studied elements on PTFE plasma sample with sG layer comparison without laser exposure, after laser exposure with energy 150 mJ/cm^2^, after exposure with laser energy 200 mJ/cm^2^ and combination of thermal stress and exposure with laser energy 200 mJ/cm^2^ (analyzed area 10 × 10 μm^2^).

The effect of laser exposure was studied, samples exposed to different laser fluences were compared, and the effect of thermal stress and subsequent exposure to the laser was investigated (see Table 2, Fig. 7). It can be stated that the excimer laser exposure induces the formation of spherical bimetallic composite particles with PTFE. As a result of thermal stress before laser exposure, the mass representation of metal elements is reduced. A higher temperature would likely be required to achieve a more dramatic chemical change. Both PTFE [48] and metal nanoparticles [49] are thermally very stable substances. Increasing the energy of the laser beam from 150 to 200 mJ/cm^2^ did not cause a significant change in the representation of individual elements. Comparison of the chemical composition of samples after exposure to a laser with an energy of 200 mJ/cm^2^ is introduced in Table 3.

**Table 2:** Comparison of the chemical composition of the sample PTFE plasma sG, also sG after exposure to a laser energy of 150 mJ/cm^2^, sG heated for 1 h 100 °C laser 150 mJ/cm^2^ and sG laser 200 mJ/cm^2^ (analyzed area 10 × 10 μm^2^).

	Elemental concentration (wt. %)				
	C	F	Ag	Au	O
PTFE plasma **sG**	11.7	25.6	10.0	52.7	0
PTFE plasma **sG** laser 150 mJ/cm^2^	19.5	49.5	5.3	25.7	0
PTFE plasma **sG** T laser 150 mJ/cm^2^	32.7	43.5	4.0	19.8	0
PTFE plasma **sG** laser 200 mJ/cm^2^	24.9	46.0	5.2	23.9	0

**Table 3:**
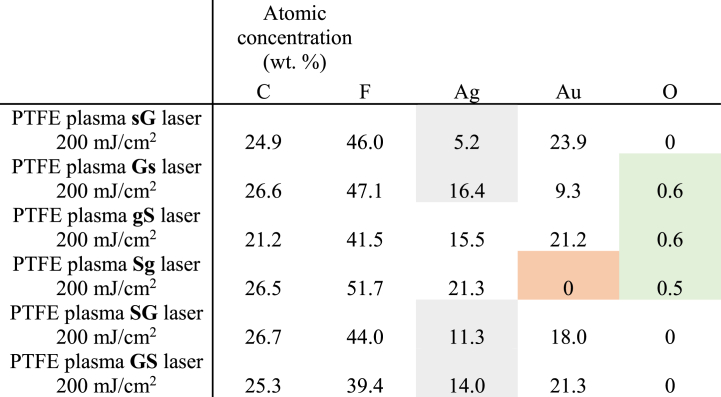
Results of EDS analysis. Comparison of the chemical composition of samples with different order and thickness of metal layers after exposure to a laser with an energy of 200 mJ/cm^2^ (analyzed area 10 × 10 μm^2^).

**Fig. 8:**
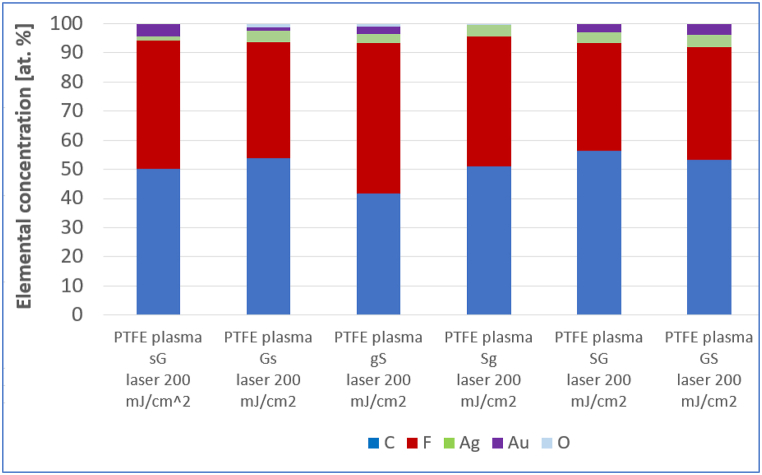
EDS analysis results, atomic representation of individual elements and % of PTFE plasma samples sG, Gs, gS, Sg, SG and GS after exposure to a laser with an energy of 200 mJ/cm^2^ (analyzed area 10 × 10 μm^2^).

The higher value of the atomic concentration of silver for silver layer deposited PTFE with a primary gold layer compared to the Ag layer on plasma-modified PTFE shows an improvement in the ability of silver nucleation on a slightly more hydrophilic substrate. In some cases, in addition to the expected elements (carbon C, fluorine F, silver Ag and gold Au), oxygen O is also present (see Fig. 8). Oxygen is present mainly in cases when a thicker silver layer was sputtered. Silver is probably subject to a slight oxidation, which will be reflected in the particular elemental composition. Silver atoms are more susceptible to oxidation than gold atoms, therefore oxygen is only found in these cases. No surface gold was noted for the PTFE plasma Sg sample. The anomaly could have been caused by “over-sputtering” of continuous Ag layer.

**Fig. 9:**
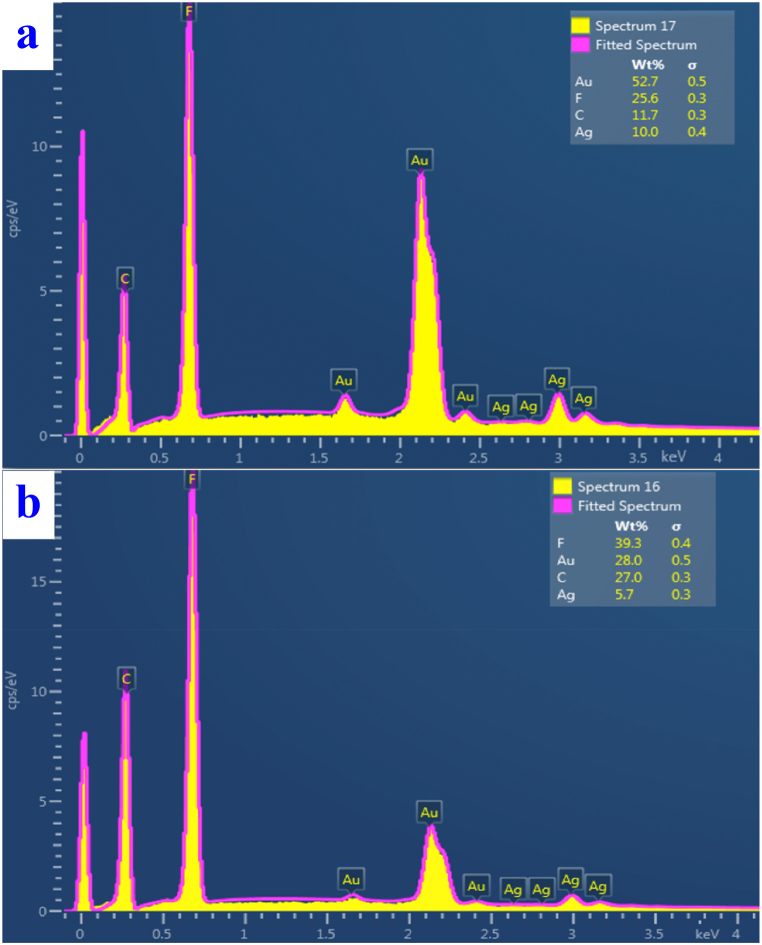
Spectra obtained by the EDS method for PTFE plasma samples with sG layers without laser exposure (a) and after exposure to an excimer laser with an energy of 200 mJ/cm^2^ (b), (analyzed area 10 × 10 μm^2^).

Fig. 9 shows the spectra obtained using EDS microscopy, here for exposure of the sample to a higher laser energy of 200 mJ cm^−2^. The spectra show a significant change in the mass representation of the Ag layer – the value dropped from 10 wt % to 5.7 wt %. There was also an almost two-fold decrease in the mass percentage of Au - from the original 52.7 wt % to 28.0 wt %. As commented earlier, the change is likely due to layer ablation associated with excimer laser exposure.

### 3.4 Surface chemistry – Infrared spectroscopy

**Fig. 10:**
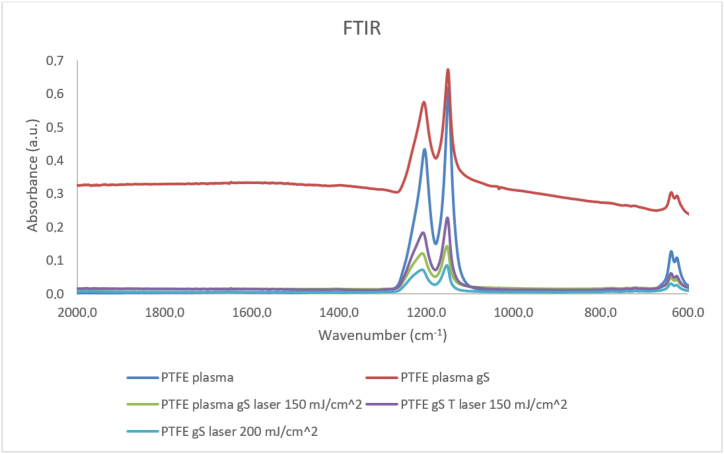
Results of FTIR measurement of PTFE plasma samples, PTFE plasma samples with gS layer, PTFE plasma with gS layer after laser exposure with laser fluence of 150 mJ/cm^2^, PTFE with gS with a combination of thermal stress (1 h 100 °C) and laser exposure with laser fluence of 150 mJ/cm^2^ and PTFE with gS layer after laser exposure with with laser fluence of 200 mJ/cm^2^.

The chemical composition was also investigated by the FTIR method (Fig. 10). Measurements were made for PTFE plasma foil, and also for PTFE sputtered with gS layers and exposed to excimer laser of different energies, as well as for a combination of thermal stress and laser exposure. The possibility of the reaction of metal particles with PTFE foil in the case of application of a relatively high laser beam energy and the formation of new asymmetric bonds was mainly studied.

The chemical composition of the PTFE plasma substrate was confirmed. A peak at approximately 1154 cm^−1^ characteristic of the symmetric valence vibration of the -CF2- group and 1210 cm^−1^ characteristic of the asymmetric valence vibration of the same bond was observed. The third peak with a wave number of approximately 644 cm^−1^ also belongs to the deformation vibration of bonds [50,51]. Peaks at similar wavelengths were observed for all sample types. As a result of the laser exposure of the samples, no new asymmetric bonds were formed, or these signals were too small.

### 3.5 Antibacterial testing

In the case of nanoparticles, two mechanisms of antibacterial affects are hypothesized. The first describes the passage of particles through the outer wall and capture on the inner wall. Adhesion to the inner membrane causes its destabilization and increases its permeability. This “flushes out” the cell's internal contents and causes death. The second mechanism assumes passage not only through the outer but also through the inner wall. The ability of silver ions to react with proteins containing sulphur or phosphorus has been proven, which leads to disruption of the DNA structure and the death of the cell [52]. In the case of bulk silver (surface effect) it is the same mechanism, so again silver ions act on specific groups of DNA, causing disruption [53]. In contrast, gold nanoparticles generally do not show toxicity to both types of bacteria [54]. However, they can be modified or activated in such a way that they are active against bacteria. The main factors that can change the antibacterial activity are size, concentration, surface modifications (coatings or various stabilizing agents) [[55], [56], [57], [58]]. There are a number of articles demonstrating the bactericidal effect of gold nanoparticles [59,60]. The main difference between the activity of silver and gold nanoparticles lies in their different willingness to oxidize. As already mentioned, the mechanism of effect of silver particles involves silver ions. A similar mechanism can also be applied to gold nanoparticles. However, gold is more stable and less willing to oxidize, and therefore is not capable of bacterial killing under normal conditions [55]. Antibacterial surfaces of bimetallic structures has been studied extensively in e.g. Refs. [[61], [62], [63], [64], [65]]. By combining silver and gold, bimetallic nanostructures can be created. Mixing will moderate the release of Ag + ions into the environment and thereby reduce toxicity. By varying the composition ratios of both metals, the degree of antibacterial effect can be regulated [31].

**Fig. 11:**
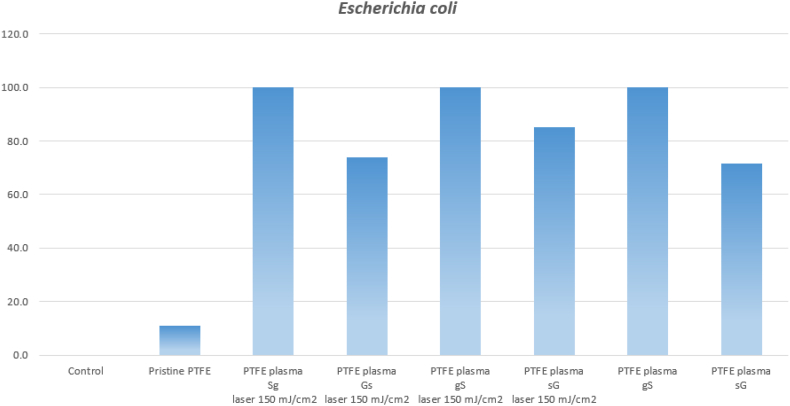
Antibacterial tests performed on *E.coli* for PTFE plasma with different combinations of metal layers, some also after laser exposure (150 mJ/cm^2^), monitoring after 24 h, the relative y-axis related to control sample is introduced (100 means full inhibition).

Selected samples were subjected to antibacterial testing. Four main combinations of samples were selected, namely PTFE plasma with sG, Sg, gS and Gs layers after exposure to a laser beam with an energy of 150 mJ/cm^2^. In order to determine the effect of the laser beam, only sputtered samples of PTFE plasma sG and gS were also studied. The samples were tested against two types of bacteria: *Escherichia coli (E. coli)* and *Staphylococcus aureus (S. aureus)*. Results were recorded after 2 and 24 h. In the case of the effect of the above samples on *E. coli*, no significant antibacterial effect was observed after 2 h. For the sake of simplicity, the results were not shown in the graph. After 24 h, a change was already visible (Fig. 11). All samples with “thicker” silver layer (Sg laser 150 mJ/cm^2^, gS laser 150 mJ/cm^2^ and gS) showed a strong antibacterial effect, thus demonstrating high antibacterial activity not only of silver nanoparticles induced by laser exposure, but also of the discontinuous deposited Ag nanostructures themselves. The result corresponds with statements from the literature mentioned above in the paragraph. The antibacterial effect was not suppressed even in the case of the sample PTFE plasma Sg, i.e. even a thin layer of gold on the deposited thick silver did not change the antibacterial activity. This effect can be explained from the point of view of surface morphology. After exposure with an excimer laser, globular structures are formed and Au/Ag/PTFE composites are also formed, which leads to the creation of an antibacterial surface even for the type of sample where the Au layer was deposited on Ag.

The samples with thin silver and thick gold layers (regardless of the order or application of the high-energy beam) were antibacterial, however, less so than the samples with a thick silver film. Strong antibacterial properties were observed for electrodeposited silver coated graphene oxide on NiTi [66]. The phenomenon is caused by the formation of bimetallic particles with a higher Au content, which is supposed to hinder the release of silver ions into the environment. The behaviour corresponds to the results from article [31], where a reduction in the release of the source of antibacterial properties, here Ag + ions, was also found in bimetallic structures with a lower Ag content. A surprising result was found for the PTFE plasma Gs sample with a laser exposure of 150 mJ/cm^2^, which was not strongly active. However, the laser probably caused the ablation of silver atoms and also the formation of a composite of both elements, probably also with PTFE, which reduced the release of Ag + ions into the environment and thus the bactericidal effect.

**Fig. 12:**
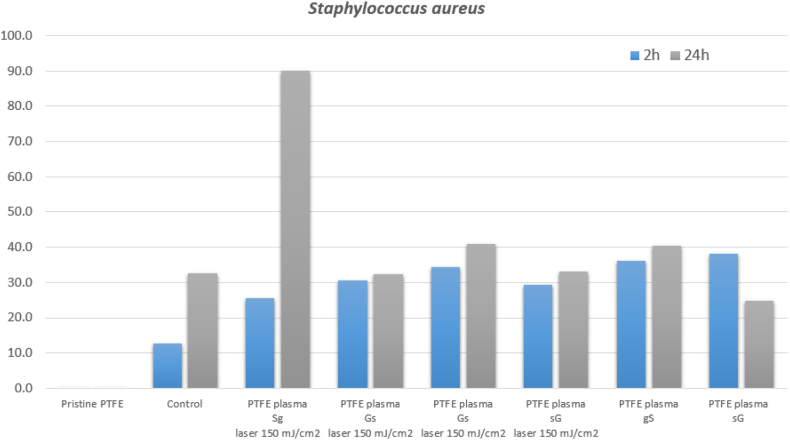
Antibacterial tests performed on *S. aureus* for PTFE plasma with different combinations of metal layers, some also after laser treatment (150 mJ/cm^2^), monitoring after 2 and 24 h. The relative y-axis related to pristine sample is introduced. (100.0 means full inhibition).

The activity against *Staphylococcus aureus* bacteria was also monitored after 2 and 24 h (Fig. 12). In this case, a decrease in the number of bacteria can be observed after only 2 h. All samples with noble metals showed similar antibacterial activity. After 24 h there was a further decrease in the number of bacteria. Except for one sample (Sg laser exposure 150 mJ/cm^2^), again all samples had a similar weak antibacterial effect. The result is surprising, especially compared to the gS laser 150 mJ/cm^2^ sample, which was expected to be larger (presence of a higher amount of Ag on the surface).

**Fig. 13:**
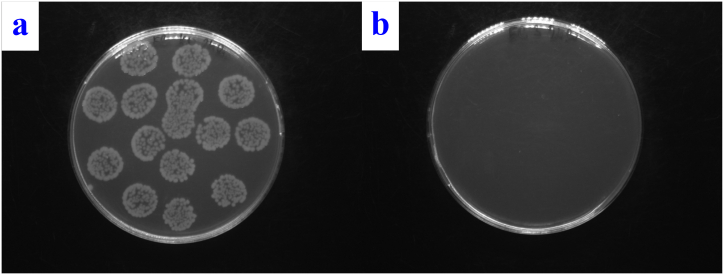
Images of *E. coli* colonies on the control sample after 24 h of incubation (a) and image of *E. coli* colonies of the PTFE plasma gS sample after laser exposure with an energy of 150 mJ/cm^2^ after 24 h of incubation (b).

**Fig. 14:**
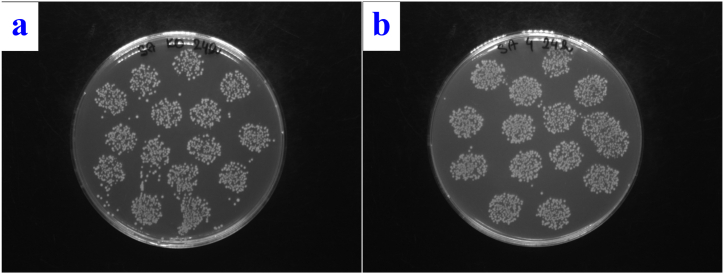
Images of *S. aureus* colonies on the control sample after 24 h of incubation (a) and *S. aureus* colonies of the PTFE plasma sG sample after laser exposure with an energy of 150 mJ/cm^2^ after 24 h of incubation (b).

The discrepancy can again be explained by the ablation of the upper silver film and the formation of a composite in the case of PTFE gS laser exposure of 150 mJ/cm^2^. In contrast, in the case of the bottom "thick” silver layer and the top thin gold layer (Sg laser exposure 150 mJ/cm^2^), composite nanoparticles are more likely to be formed. The demonstrably strongest effect against both types of bacteria was observed for sample Sg laser exposure of 150 mJ/cm^2^ (for photos see Fig. 13, Fig. 14).

The limitations of the studied surface treatment techniques are based mostly on the applied surface exposure. If the higher laser power is applied, it may lead to surface ablation instead of nanostructure formation. We also have performed some experiments with laser fluences above 500 mJ cm^−2^, where an ablation effect of both metal nanolayers and polymer took place. It would be possible to increase the primary thickness of the deposited metals, which would lead to the nanoparticle formation also for higher laser fluences. As one of the future perspective, we plan to conduct the experiments for different metals, such is Cu. The exposure of Cu nanolayers, Cu nanoparticle formation or the combination with other metals could lead also to excellent increase of surface antibacterial properties.

### 3.6 Replication of bimetallic pattern

**Fig. 15:**
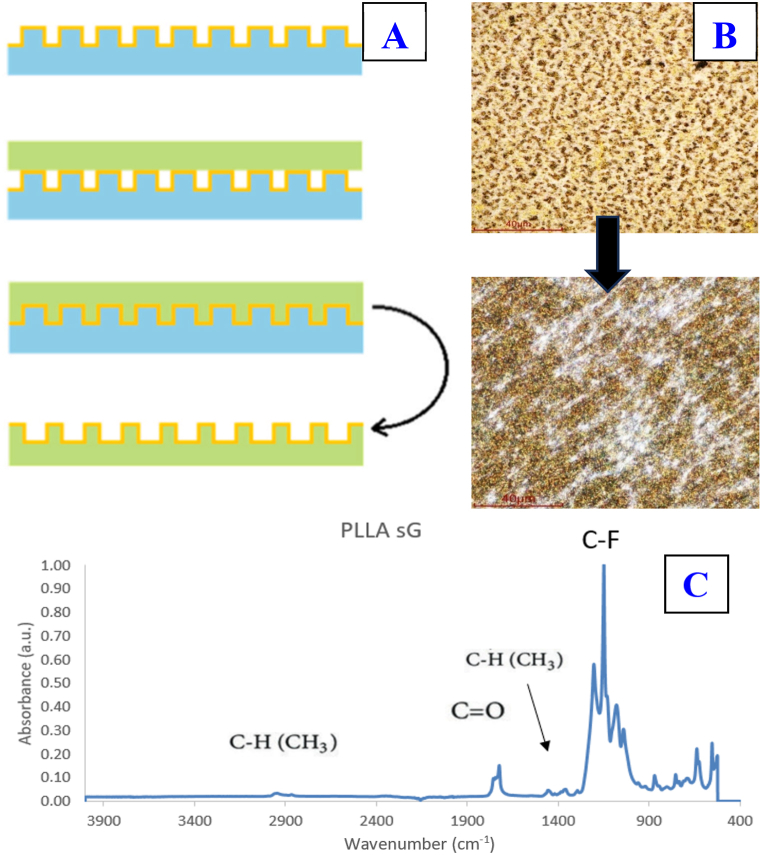
Schematic of the transfer of surface morphology and also metal structure that occurs during replication (A) [67]. Comparison of the surface morphology studied by the LCM method of the surface of the original sample PTFE plasma sG laser exposure with an energy of 150 mJ/cm^2^ (up) and PLLA replica (down) (B).

The surface of the selected samples was replicated into a polylactic acid substrate. Specifically, PTFE plasma samples sG, Sg, gS and Gs were replicated, all also after exposure to a 150 mJ/cm^2^ laser. In this way, not only the fiber pattern of the PTFE foil was transferred, but also the complete transfer of the metal pattern from one polymer to another. The process can be imagined according to the following Fig. 15.

The value of the contact angle of the unmodified PLLA film is in the range of 75–85° [68]. The value we found was 79.3 ± 0.9°. After replicating the PTFE surface with deposited metal layers on the PLLA film, there was a large increase in the hydrophobic character of the polymer. This is due to a change in the surface morphology, and in the replication (transfer) not only of the metal structure, but also of the part of the fibers of the PTFE foil/PTFE-noble metal composite (see Fig. 15A and B). This assumption will also be further confirmed. The order and height of the metal structures on the PLLA surface has no significant effect on the wettability of the sample. The transferred PTFE therefore has a higher influence and suppresses the hydrophilic character of the metal layers.

From the images taken on the LCM microscope, the transfer of the pattern to the PLLA foil is evident, even with the lower fibrous structure of PTFE (Fig. 15B). The white fibrils on the surface of the sample correspond to PTFE nanofibers. The assumption is confirmed by the chemical analysis of the surface presented in the following chapter. The surface chemistry was studied by the FTIR method (Fig. 15C). A sample of PLLA was measured, followed by PLLA with a replicated surface. The measurement was performed on a selected group of samples, a sample with a replicated surface made of PTFE plasma sG laser exposure 150 mJ/cm^2^ was selected for demonstration. When measuring PLLA by FTIR analysis, absorption peaks can be observed at the following wavenumber values: C-H bond vibrations are responsible for the peak in the area around 3000 cm^−1^, in the area around 1748 cm-1 C=O vibrations are responsible, and -CH_3_ groups vibrate around 1453 cm^−1^. Three peaks in the regions of 1128, 1082 and 1043 cm^−1^ indicate symmetric C-O-C vibrations [69]. The same absorption peaks were observed for the PLLA substrate measured in this work. The result of measuring PLLA with a replicated surface of PTFE plasma sG laser exposure with an energy of 150 mJ/cm^2^ is interesting. Peaks characteristic of C-H vibrations (3000 cm^−1^), also C=O vibrations (1748 cm^−1^) and the deformation region of -CH3 groups (1453 cm^−1^) can be observed. The absorption peak of C-O-C vibrations is suppressed and replaced by peaks that are characteristic of C-F bonds. The measurement result confirms the previous idea about the transfer of parts of PTFE nanofibers from the foil during replication.

## 4 Conclusions

Bimetallic Ag/Au nanoclusters were prepared on the polytetrafluoroethylene nanofibrous material by high energy excimer laser exposure. The materials were exposed with an excimer laser with laser fluences of 150 or 200 mJ/cm^2^. Selected samples underwent also heat treatment. The influence of the thickness of the prepared layers as well as their order on the target properties of the prepared structures was investigated. Selected bimetallic structures were replicated into a poly-L-lactic acid polymer. Laser exposure led to an increase of contact angle for all types of samples, close to the superhydrophobicity values. The exposure of the PTFE substrate with sputtered Ag and Au layers led to formation of the Ag/Au-PTFE composite. The study of surface morphology by SEM and AFM methods confirmed the formation of globular Ag/Au-PTFE nanostructures after laser exposure. The study of the chemical composition using the SEM/EDS method revealed the formation of bimetallic structures. Antibacterial tests demonstrated the bactericidal effect of all types of samples against two types of bacteria: E. coli and S. aureus. The strongest toxicity towards both types of bacteria was shown by the PTFE plasma Sg sample after laser exposure with an energy of 150 mJ/cm^2^. The pattern of PTFE plasma foils with Ag/Au layers after laser exposure was replicated in lactic acid polymer. After replication, the contact angle value increased to around 140° due to the increase in surface roughness and also the transfer of not only metal layers but also PTFE chains.

## CRediT authorship contribution statement

**Petr Slepička:** Writing – original draft, Supervision, Funding acquisition. **Tereza Vašinová:** Investigation. **Bára Frýdlová:** Investigation. **Anna Kutová:** Investigation. **Šárka Havlíčková:** Investigation. **Václav Švorčík:** Formal analysis, Data curation. **Nikola Slepičková Kasálková:** Writing – review & editing, Validation, Methodology.

## Data availability statement

The data presented in this study are available at https://doi.org/10.5281/zenodo.14725044.

## Declaration of competing interest

The authors declare that they have no known competing financial interests or personal relationships that could have appeared to influence the work reported in this paper.
